# 5hmC Level Predicts Biochemical Failure Following Radical Prostatectomy in Prostate Cancer Patients with ERG Negative Tumors

**DOI:** 10.3390/ijms20051025

**Published:** 2019-02-27

**Authors:** Gitte Kristensen, Siri H. Strand, Martin Andreas Røder, Kasper Drimer Berg, Birgitte Grønkær Toft, Søren Høyer, Michael Borre, Karina Dalsgaard Sørensen, Klaus Brasso

**Affiliations:** 1 Copenhagen Prostate Cancer Center, Department of Urology, Rigshospitalet, University of Copenhagen, 2100 Copenhagen, Denmark; andreasroder@gmail.com (M.A.R.); kasperdrimerberg@gmail.com (K.D.B.); klaus.brasso@regionh.dk (K.B.); 2 Department of Molecular Medicine, Aarhus University Hospital, 8200 Aarhus, Denmark; siri.strand@clin.au.dk (S.H.S.); kdso@clin.au.dk (K.D.S.); 3 Department of Pathology, Rigshospitalet, University of Copenhagen, 2100 Copenhagen, Denmark; birgitte.groenkaer.toft@regionh.dk; 4 Department of Pathology, Aarhus University Hospital, 8200 Aarhus, Denmark; soren.hoyer@gmail.com; 5 Department of Urology, Aarhus University Hospital, 8200 Aarhus, Denmark; borre@clin.au.dk

**Keywords:** prostate cancer, predictive biomarkers, radical prostatectomy, 5hmC, ERG

## Abstract

This study aimed to validate whether 5-hydroxymethylcytosine (5hmC) level in combination with ERG expression is a predictive biomarker for biochemical failure (BF) in men undergoing radical prostatectomy (RP) for prostate cancer (PCa). The study included 592 PCa patients from two consecutive Danish RP cohorts. 5hmC level and ERG expression were analyzed using immunohistochemistry in RP specimens. 5hmC was scored as low or high and ERG was scored as negative or positive. Risk of BF was analyzed using stratified cumulative incidences and multiple cause-specific Cox regression using competing risk assessment. Median follow-up was 10 years (95% CI: 9.5–10.2). In total, 246 patients (41.6%) had low and 346 patients (58.4%) had high 5hmC level. No significant association was found between 5hmC level or ERG expression and time to BF (*p* = 0.2 and *p* = 1.0, respectively). However, for men with ERG negative tumors, high 5hmC level was associated with increased risk of BF following RP (*p* = 0.01). In multiple cause-specific Cox regression analyses of ERG negative patients, high 5hmC expression was associated with time to BF (HR: 1.8; 95% CI: 1.2–2.7; *p* = 0.003). In conclusion, high 5hmC level was correlated with time to BF in men with ERG negative PCa, which is in accordance with previous results.

## 1. Introduction

Prognostication of outcome for prostate cancer (PCa) is currently based on clinicopathological parameters [1,2,3]. Several studies have shown that active surveillance of patients with low risk disease is safe, but not without risk of progression [4,5]. In contrast, some localized PCas treated with a curative intent turn out to be more aggressive than predicted by the clinicopathological variables [6]. Applying biomarkers that reflect the biology of the tumor could potentially be a supplementary approach to the clinicopathological variables, and refine prognostication of PCa patients. Despite the enormous number of potential biomarkers reported in the literature and the clear clinical need for better prognostication and individualized treatments, no biomarkers, except for a prostate-specific antigen (PSA), are used routinely. One repeating issue is the lack of validation studies of potential novel biomarkers. 

Approximately 40%–60% of PCa patients carry the *TMPRSS2*-*ERG* gene fusion resulting in ERG protein expression [7]. Even though there is conflicting evidence whether ERG could be a biomarker for PCa aggressiveness, several studies indicate that distinct molecular mechanisms are at play in ERG negative vs. ERG positive tumors; thus, ERG in combination with other biomarkers might be a valuable biomarker for PCa aggressiveness [8,9,10].

Epigenetic modifications of the human genome have been shown to be important at all stages of carcinogenesis and progression [11], particularly dysregulation in methylation has been shown to have promising potential as a marker for cancer aggressiveness [12]. The DNA methylation variant 5-hydroxymethylcytosine (5hmC) plays an important role in epigenetic reprogramming and regulation of tissue-specific gene expression [13,14]. In the first large-scale study of 5hmC immunoreactivity in PCa patients, we have previously shown that high immunoreactivity of 5hmC is a significant adverse predictor of biochemical failure (BF) following radical prostatectomy (RP) in patients with ERG negative PCas [15,16]. Thus, the combination of 5hmC level and ERG protein expression might hold predictive value as biomarkers for PCa aggressiveness [15,16]. However, to facilitate implementation in the clinic the results need to be validated.

In the present study, we aimed to investigate the generalizability of the previously reported predictive value of 5hmC level in combination with ERG expression by adding another large-scale consecutive RP cohort from another institution. Our results indicate that 5hmC level can predict BF following RP for PCa in patients with ERG negative tumors, which is consistent with previous results. 

## 2. Results

**Figure 1:**
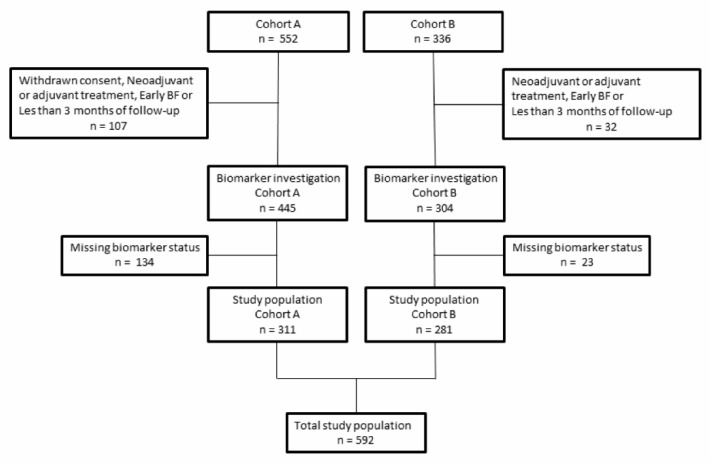
Flowchart of patients who met study inclusion/exclusion criteria.

This study included 592 patients who underwent RP for PCa (Figure 1). Pre- and postoperative clinicopathological parameters are outlined in Table 1. The median PSA was 10.6 µg/L (interquartile range: 7.1–16.0). A total of 66.3% had pT2 PCa and 87.5% of patients had RP Gleason score (GS) ≤7. The median follow-up time following RP was 10 years (95% CI: 9.5–10.2).

Immunohistochemical (IHC) analysis of tumor tissue showed that 246 (41.6%) patients had a low 5hmC level while 346 (58.4 %) patients had a high 5hmC level. Furthermore, 238 (40.2%) were ERG negative and 354 (59.8%) were ERG positive. We found a significant association between 5hmC level and lower age at RP (*p* = 0.003), lower clinical tumor-stage (*p* = 0.03), lower biopsy GS (*p* = 0.05), lower RP GS (*p* = 0.001) and positive ERG expression (*p* < 0.0001). In contrast, no association between 5hmC level and preoperative PSA (*p* = 0.7), pathological tumor-stage (*p* = 0.5) and surgical margin status (*p* = 0.8) was found (Table 1).

### Biochemical Failure

**Figure 2:**
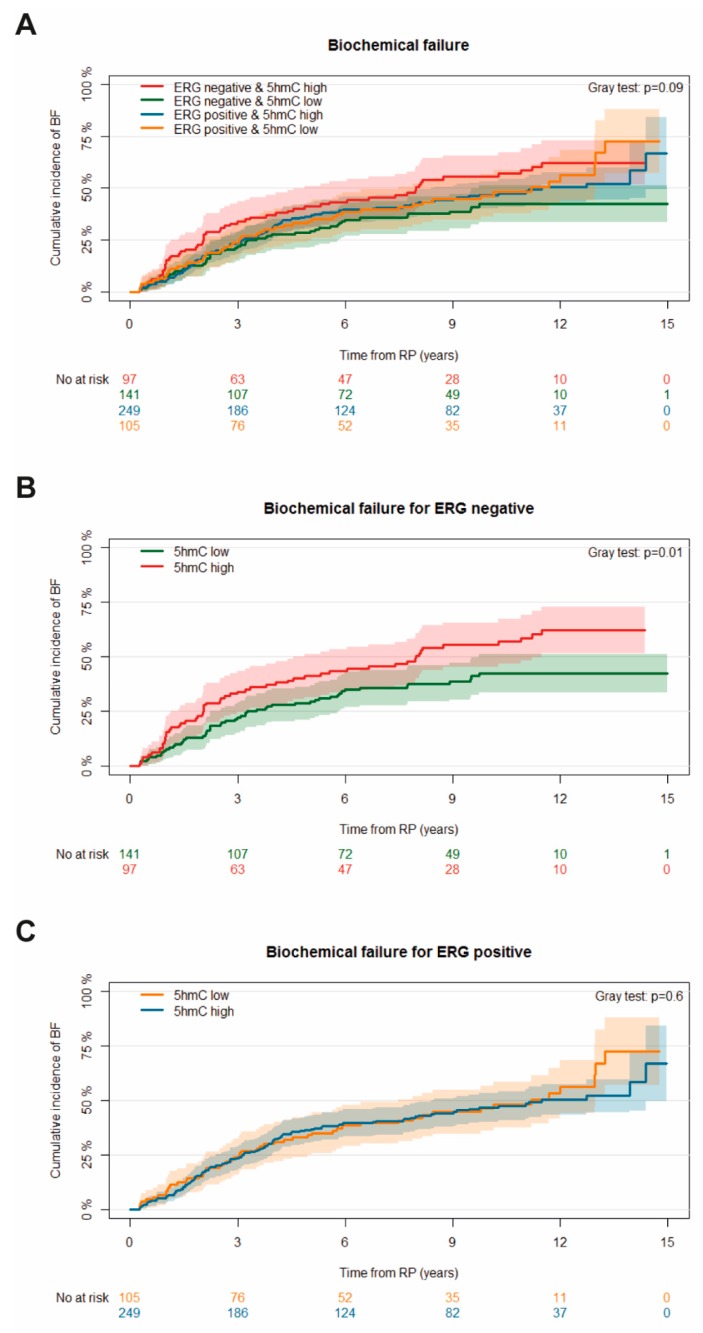
The cumulative incidence of biochemical failure (BF) following radical prostatectomy (RP). Competing events are death without BF. Patients are stratified according to (**A**) biomarker status and 5hmC level for (**B**) ERG negative and (**C**) ERG positive, respectively. The *p*-values for Gray’s test are added.

The 10-year cumulative incidence of BF was 47.1% (95% CI: 42.8–51.4). There was no association between 5hmC level or ERG expression and time to BF (*p* = 0.2 and *p* = 1.0, respectively) (Supplementary Figure S1). Moreover, no association between 5hmC level and time to BF was found when stratifying for ERG expression (*p* = 0.09) (Figure 2A). However, in ERG negative patients, the 10-year cumulative incidence of BF was 42.4% (95% CI: 33.6–51.2) in the 5hmC low group compared to 55.4% (95% CI: 45.2–65.6) in the 5hmC high group (*p* = 0.01) (Figure 2B). In contrast, no association between 5hmC level and time to BF was found in ERG positive patients (*p* = 0.6) (Figure 2C).

**Figure 3:**
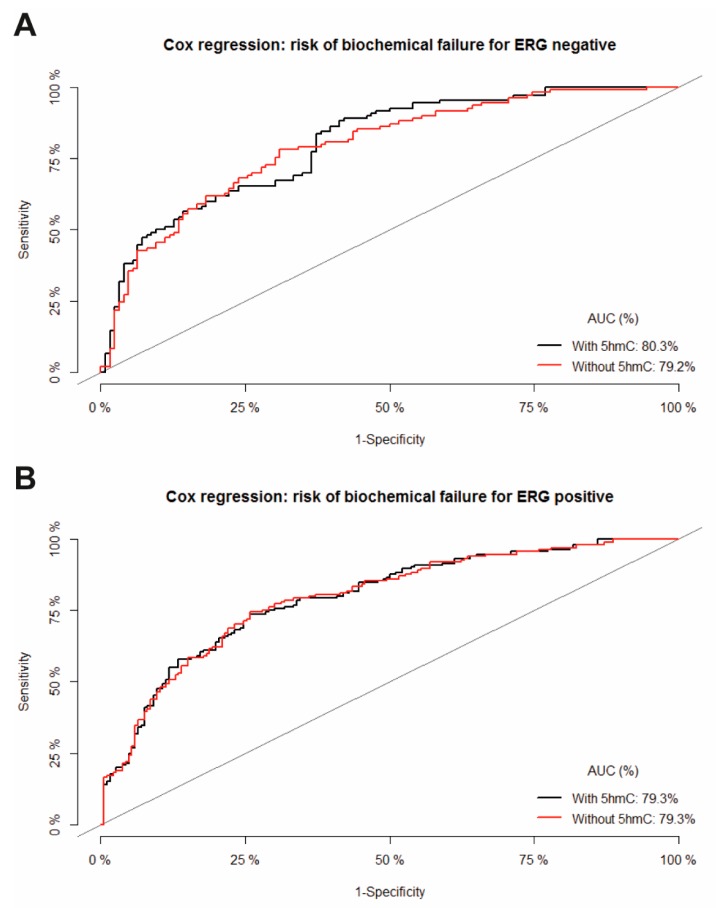
Receiver operating characteristic (ROC) curves for the multivariate cause-specific Cox regression model with and without 5hmC level for predicting biochemical failure of (**A**) ERG negative and (**B**) ERG positive patients. Area under the receiver operating characteristic curve (AUC) are added.

In multiple cause-specific Cox regression analyses high 5hmC level was an independent predictor of BF following RP in patients with ERG negative tumors (HR: 1.8; 95% CI: 1.2–2.7; *p* = 0.003) (Table 2A), whereas 5hmC level did not have a predictive value in terms of BF in patients with ERG positive tumors (Table 2B). The sensitivity and specificity of the models with and without 5hmC for predicting BF are presented in the receiver operating characteristic (ROC) plot for ERG negative (Figure 3A) and ERG positive (Figure 3B) tumors. The areas under the curve (AUC) value for the model without 5hmC was 79.2% which increased to 80.3% with the addition of 5hmC level in the model for ERG negative tumors. There was no difference in the AUC for the models with and without 5hmC level of BF for ERG positive tumors.

## 3. Discussion

Treatment of PCa with RP is intended to be curative, but a long-term follow-up indicates that approximately 40% will experience BF and 15% will progress to a metastatic disease [17,18]. Several nomograms have been established to estimate the risk of progression [1,2,3], but all lack accuracy. Therefore, new tools, including the identification of novel biomarkers for more precise prognostication of PCa aggressiveness, are essential.

Epigenetic modifications regulate gene expression by either DNA methylation, histone modifications or microRNA. Changes in epigenetic modifications may result in inaccurate activation or inhibition of signaling pathways leading to the development of cancer [11]. It is well established that PCa has a low number of somatic gene mutations [19]. Consequently, epigenetic alterations are considered hallmarks for PCa development and progression [20,21]. Particularly, dysregulation in DNA methylation has shown to be important in the initiation and progression of cancer [11,22]. DNA methylation at the 5 position of cytosine (5mC) is important for various biological as well as pathological processes [11]. 5mC can be converted to 5hmC by the ten-eleven translocation (TET) family of DNA hydroxylases [13]. Several studies have indicated that changes in DNA methylation may reflect PCa aggressiveness [16,21,23,24]. Furthermore, 5hmC has previously been associated with several cancer types [12,25,26] including PCa [14,15,16,25,26,27], but so far the only study that analyzed the association between 5hmC level in tumor tissue and PCa aggressiveness was our previous study [15,16].

Since the combination of 5hmC level and ERG expression was found to be associated with BF following RP for PCa, we aimed to validate the biomarkers in the present study by including another RP cohort (cohort B) and extending the follow-up by three years in the original cohort (cohort A). In the present study, we found that 5hmC level was associated with RP GS and ERG expression. Furthermore, consistent with our previous results, we confirmed that a high 5hmC level predicted BF in patients with ERG negative tumors [15,16]. Thus, our findings suggest that 5hmC level is predictive of BF in patients with ERG negative tumors, including 5hmC level in the predictive model only increased the AUC by 0.7%.

Two small-scale studies have previously shown that 5hmC level was reduced in prostate tumor tissue when compared to normal prostate tissue [25,26]. Haffner MC et al. analyzed 30 PCa tissue samples and found that 5hmC immunoreactivity was not associated with tumor grade and stage [25]. In accordance, we did not find an association between 5hmC level and pathological tumor-stage, but in contrast, we demonstrated a significant negative association between 5hmC level and RP GS. This difference from other studies might be a consequence of power. A high 5hmC level has previously been shown to be associated with a poor outcome in patients with acute myeloid leukemia [28]. In contrast to that, a low 5hmC level has been found to be associated with a poor outcome in various cancers including breast cancer [29], malignant melanoma [30], non-small cell lung cancer [31], renal cell carcinoma [32] and cervical squamous cell cancer [33]. When including the results of the present study it seemed reasonable to suggest that different mechanisms are at play in different types of cancer. An explanation could be that different phenotypic effects of epigenetic regulation are influenced by the global level of specific DNA methylations and their genomic distribution in the different cell types, as well as by specific patterns of genomic aberrations in cancer types. 

We previously demonstrated that the presence of ERG can predict progression of early and low-risk PCa [34]. In contrast, ERG expression does not seem to add any predictive information regarding the risk of BF following RP [35], which is consistent with our results. However, in line with previous studies, we demonstrated that ERG status can be used to distinguish between different PCa molecular subtypes [8,9,10]. Moreover, our results contribute to the perception that ERG stratification is important in predicting PCa outcome and when investigating new biomarkers for PCa aggressiveness. 

Despite enormous research activity, no IHC based tissue biomarker is routinely used in the daily clinical practice of handling PCa patients. One likely explanation is the lack of validation studies, which are needed to verify the accuracy of the biomarker [36]. Another issue is the missing consensus regarding the selection of antibodies and standardization of the IHC technique. Finally, scoring of the IHC staining is done semi-quantitatively, and known to be associated with inter- and intra-observer variation, which may limit its reproducibility [37]. As our previous study was the first large-scale investigation of 5hmC immunoreactivity in combination with ERG in PCa, it is a strength of the present study that it seeks to validate our results in another study population, as the addition of a cohort from another institution increases the generalizability of the results across study populations. Furthermore, it is a strength that we used the same antibody and the same protocol for the IHC staining, and that the scoring of 5hmC immunoreactivity was done in accordance with our first study. Although the scoring of the IHC staining was done by different observers for the two cohorts, the observer of cohort B had thorough instructions from one of the observers of cohort A. Since inter-observer agreement was high in our first publication [15], we assessed that one observer for cohort B would be sufficient. Moreover, the study cohorts were two consecutive series of men who underwent RP with curative intent and long-term follow-up, and few lost-to-follow-up. Finally, even though the cohorts were historical, the indication for RP has not changed through the study period and the RP GS was updated according to the International Society of Urological Pathology (ISUP) 2005 Gleason grading system for both cohorts [38].

Our study has some limitations, besides those related to its retrospective nature. First, only a small fraction of the tumor tissue from the RP specimen was evaluated for 5hmC level and ERG expression, and the fact that PCa is known to have intratumoral heterogeneity can hamper the reliability of the biomarker status. Second, even though GS 3 + 4 and GS 4 + 3 are generally accepted as two different risk groups, we analyzed them as a single group because we did not have access to primary and secondary Gleason grade for all the patients. Third, we used BF as an endpoint, acknowledging that BF is only a surrogate endpoint for PCa aggressiveness. Whether 5hmC in combination with ERG can predict a more clinically relevant endpoint, such as metastasis and PCa specific death, needs to be investigated. Finally, we used antibodies from two different companies to analyze ERG expression in the two cohorts, but as the antibodies are from the same clone EPR3864 we expect that they bind equally. Furthermore, investigation of ERG protein expression by the antibody clone, EPR3868, has previously shown to be specific for ERG gene fusion status [39,40,41]. 

Based on our findings it can be hypothesized that 5hmC level in combination with ERG status can be used to select patients who would benefit more from a closer follow-up and/or early adjuvant or salvage therapy. Furthermore, it can be speculated whether the combination of 5hmC level and ERG expression can be used to select the most eligible low-risk PCa patients for an initial observational strategy. Logically, further research, including more patients and other outcomes, is needed to determine the true predictive as well as prognostic value of 5hmC in PCa. 

## 4. Materials and Methods

### 4.1. Patient Cohort

The study included two historical consecutive RP cohorts from two independent urological departments at university hospitals in Denmark (Copenhagen & Aarhus). We used tissue microarrays (TMAs) generated from formalin-fixed paraffin-embedded RP specimens from both tumor and normal prostate tissue. The cohort included 552 men who underwent RP from 1998 until 2009 at the Department of Urology, Aarhus University Hospital, Aarhus, Denmark (cohort A), and 336 men who underwent RP from 2002 until 2005, at the Department of Urology, Copenhagen University Hospital, Rigshospitalet, Copenhagen, Denmark (cohort B) (Figure 1). Production of the TMAs and collection of the clinicopathological data has previously been described in detail [15,42]. The study was approved by the Danish National Committee on Health Research Ethics (Journal no.: 2000-0299, 28 March 2017 and H-6-2014-111, 6 February 2015) and The Danish Data Protection Agency (file#2013-41-2041 and file#2006-1-6256). 

Clinical and pathological information were collected from patient records. The RP GS was reclassified according to the ISUP 2005 Gleason grading system [38]. BF was defined as PSA ≥ 0.2 ng/mL. Survival was specified using the Danish Central Person Registry that is updated daily. Patients were excluded if neoadjuvant endocrine treatment was administrated, length of follow-up was less than 3 months, BF occurred within the first 3 months following RP, or if malignant tissue was not present in the TMA (Figure 1).

### 4.2. Immunohistochemistry

Freshly cut sections of each TMA block were used for IHC staining for 5hmC (1:1000 dilution; anti-5hmC 39769 Rabbit Polyclonal antibody, Active Motif, Carlsbad, CA, USA) and ERG (cohort A: anti-ERG clone EPR3864 Rabbit Monoclonal Antibody, Epitomics, Burlingame, CA, USA, and cohort B: anti-ERG clone EPR3864 Rabbit Monoclonal Antibody, Roche Ventana, Indianapolis, IL, USA), as previously described in detail [15,42]. A separate hematoxylin & eosin (HE) stained section was used for validation of the presence of cancer in each core. 

**Figure 4:**
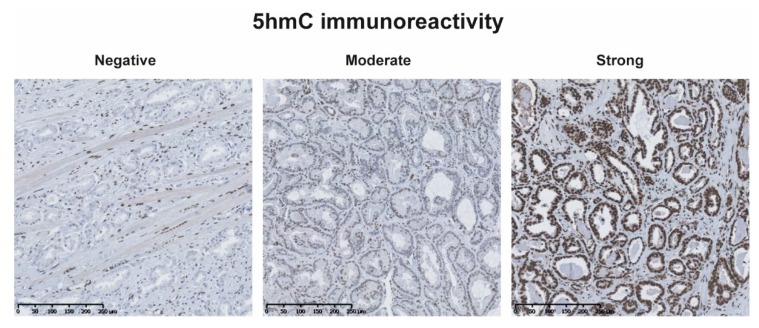
IHC 5hmC staining in representative prostate cancer samples showing negative, moderate and strong immunoreactivity.

Whole field inspection of 5hmC and ERG immunoreactivity for each TMA core was done by two independent observers for cohort A (SHS, SH) [15] and one observer (GK) for cohort B. For each TMA core, nuclear 5hmC immunoreactivity in malignant prostate epithelial cells was evaluated and scored for the predominant intensity and categorized as 0 = negative, 1 = moderate or 2 = strong (Figure 4) [15]. As each patient had multiple cores, and a mean 5hmC score (∑ intensity for each core/number of cores) was calculated for each patient. Patients with a mean 5hmC score ≤1 were classified as having a low 5hmC level and patients with a mean 5hmC score >1 were classified as having a high 5hmC level. Nuclear ERG immunoreactivity in malignant prostate epithelial cells was evaluated and scored as negative if all cores were negative, and positive if any of the cores were positive [15,42]. The assessment of the biomarkers was blinded to study end-points. 

### 4.3. Statistics

Association of 5hmC expression in tumor tissue and clinicopathological variables was analyzed using the χ^2^-test for categorical variables and the Mann-Whitney U test for continuous variables. The median time of follow-up was calculated using the reverse Kaplan-Meier method [43]. Follow-up was calculated until April 2018 for cohort A and July 2017 for cohort B. 

Cumulative incidences of BF were analyzed using the Aalen-Johansen method for competing risks. Death before BF was treated as a competing event. Gray’s test was used to assess differences in the cumulative incidence of BF between biomarker subgroups [44]. Uni- and multivariate cause-specific Cox proportional hazard regression models were performed for risk of BF, with results presented as hazard ratios (HR) and a 95% confidence interval (CI). The analysis included age at RP, log2-transformed PSA, pT-stage, RP GS, surgical margin status, ERG expression and 5hmC level. The overall ability of the models to predict BF was analyzed using the ROC curve, generated for the multivariate cause-specific Cox regression models with and without 5hmC level, and quantified using the AUC.

All tests were two-sided and *p* < 0.05 was considered as statistically significant. All statistical analyses were performed using SPSS (software version 22; IBM) or R (R Development Core Team, Vienna, Austria).

## 5. Conclusions

In conclusion, we found that a high 5hmC level was associated with BF in patients with ERG negative PCa, while 5hmC level did not have a significant predictive value in ERG positive PCa. Our findings suggest that 5hmC level in combination with ERG status holds potential as a biomarker for PCa aggressiveness. 

## Figures and Tables

**Table 1:** Baseline characteristics of the study cohort.

Baseline Characteristic of the Study Cohort	Study Population *n* = 592	5hmC Low *n* = 246	5hmC High *n* = 346	*p*-Value
Age at baseline, years, median (IQR)	63.2 (59.5–67.0)	64.1 (60.3–67.7)	62.5 (59.0–66.6)	0.003 *
PSA, µg/L, median (IQR)	10.6 (7.1–16.0)	10.9 (7.2–16.1)	10.6 (7.1–16.0)	0.7 *
Clinical T-stage				0.03 **
cT1	161 (27.2%)	53 (21.5%)	108 (31.3%)	
cT2	423 (71.6%)	190 (77.2%)	233 (67.5%)	
cT3	7 (1.2%)	3 (1.2%)	4 (1.2%)	
Missing	1	0	1	
Biopsy Gleason score				0.05 **
≤6	361 (69.4%)	133 (63.6%)	228 (73.3%)	
7	125 (24.0%)	58 (27.8%)	67 (21.5%)	
8–10	34 (6.5%)	18 (8.6%)	16 (5.1%)	
Missing	72	37	35	
Pathological T-stage				0.5 **
pT2	392 (66.3%)	167 (67.9%)	225 (65.2%)	
pT3-4	199 (33.7%)	79 (32.1%)	120 (34.8%)	
Missing	1	0	1	
Radical prostatectomy Gleason score				0.001 **
≤6	202 (34.1%)	64 (26.0%)	138 (39.9%)	
7	316 (53.4%)	141 (57.3%)	175 (50.6%)	
8–10	74 (12.5%)	41 (16.7%)	33 (9.5%)	
Margin status				0.8 **
R−	329 (56.0%)	135 (55.3%)	194 (56.6%)	
R+	258 (44.0%)	109 (44.7%)	149 (43.4%)	
Missing	5	2	3	
ERG				<0.0001 **
Negative	238 (40.2%)	141 (57.3%)	97 (28.0%)	
Positive	354 (59.8%)	105 (42.7%)	249 (72.0%)	

**Abbreviations:** IQR: inter quartile range; PSA: prostate specific antigen. * Mann-Whitney U test, ** χ^2^-test.

**Table 2:** Uni- and multivariate cause-specific Cox regression of biochemical failure.

**A: For patients with ERG negative tumors**				
**For Patients with ERG Negative Tumors**	**Univariate analysis**		**Multivariate analysis**	
	**HR (95% CI)**	***p*-value**	**HR (95% CI)**	***p*-value**
5hmC				
Low	REF		REF	
High	1.6 (1.1–2.4)	*0.01*	1.8 (1.2–2.7)	0.003
Age at RP				
For 5-yr difference	1.0 (0.9–1.2)	*0.7*	1.0 (0.9–1.2)	0.8
PSA				
For 2-fold difference	1.7 (1.4–2.2)	*<0.0001*	1.4 (1.1–1.8)	0.002
Pathological T-stage				
pT2	REF		REF	
pT3-4	3.1 (2.1–4.5)	*<0.0001*	1.9 (1.2–2.8)	0.004
RP Gleason score				
≤6	REF		REF	
7	2.5 (1.4–4.5)	*0.003*	2.2 (1.2–4.0)	0.01
8–10	5.4 (2.8–10.2)	*<0.0001*	4.6 (2.3–9.0)	<0.0001
Margin status				
R−	REF		REF	
R+	2.1 (1.5–3.1)	*<0.0001*	1.5 (1.0–2.3)	0.04
**B: For patients with ERG positive tumors**				
**For Patients with ERG Positive Tumors**	**Univariate analysis**		**Multivariate analysis**	
	**HR (95% CI)**	***P*-value**	**HR (95% CI)**	***P*-value**
5hmC				
Low	REF		REF	
High	0.9 (0.7–1.3)	0.6	1.0 (0.7–1.4)	0.9
Age at RP				
For 5-yr difference	1.1 (0.9–1.3)	0.3	0.9 (0.8–1.1)	0.5
PSA				
For 2-fold difference	1.7 (1.4–2.1)	<0.0001	1.4 (1.2–1.7)	0.0002
Pathological T-stage				
pT2	REF		REF	
pT3-4	3.0 (2.2–4.1)	<0.0001	1.9 (1.3–2.7)	0.0003
RP Gleason score				
≤6	REF		REF	
7	2.9 (2.0–4.2)	<0.0001	2.0 (1.4–3.0)	0.0004
8–10	5.7 (3.3–9.7)	<0.0001	3.6 (2.0–6.4)	<0.0001
Margin status				
R−	REF		REF	
R+	2.4 (1.7–3.3)	<0.0001	1.6 (1.1–2.2)	0.007

Abbreviations: CI: Confidence interval; HR: hazard ratio; PSA: prostate specific antigen; REF: reference; RP: radical prostatectomy.

## Supplementary Materials

Supplementary materials can be found at https://www.mdpi.com/1422-0067/20/5/1025/s1.
