# Supplementary material for: 5hmC Level Predicts Biochemical Failure Following Radical Prostatectomy in Prostate Cancer Patients with ERG Negative Tumors
Source: Int J Mol Sci. 2019 Feb 27;20(5):1025. doi: 10.3390/ijms20051025 (PMC6429366; doi:10.3390/ijms20051025)
Supplement: Supplementary file 1 [file ijms-20-01025-s001.pdf]

Supplementary Materials:

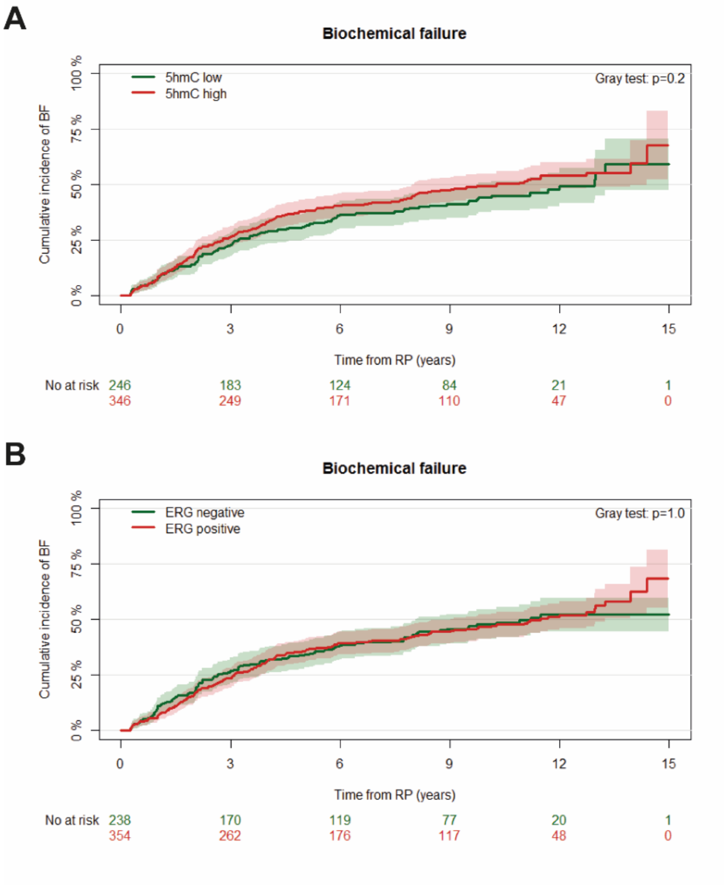

Figure S1. The cumulative incidence of biochemical failure (BF) following radical prostatectomy (RP). Competing events are death without BF. Patients are stratified according to (**A**) 5hmC level and (**B**) ERG expression. The *p*-values for Gray's test are added.
